# Burden of mortality linked to community-nominated priorities in rural South Africa

**DOI:** 10.1080/16549716.2021.2013599

**Published:** 2022-01-21

**Authors:** Pyry Mattila, Justine Davies, Denny Mabetha, Stephen Tollman, Lucia D’Ambruoso

**Affiliations:** a South Karelia Social and Health Care District (Eksote), Finland; b Institute of Applied Health Research University of Birmingham, UK; c Aberdeen Centre for Health Data Science (ACHDS), Institute of Applied Health Sciences, School of Medicine, Medical Sciences and Nutrition, University of Aberdeen, Aberdeen, Scotland; d MRC/Wits Rural Public Health and Health Transitions Research Unit (Agincourt), School of Public Health, Faculty of Health Sciences, University of the Witwatersrand, Johannesburg, South Africa; e International Network for the Demographic Evaluation of Populations and Their Health (INDEPTH), Accra, Ghana; f Department of Epidemiology and Global Health, Umeå University, Umeå, Sweden; g Public Health, National Health Service, Grampian, Scotland, UK

**Keywords:** Community participation, Participatory action research, Verbal Autopsy, Global Burden of Disease

## Abstract

**Background:**

Community knowledge is a critical input for relevant health programmes and strategies. How community perceptions of risk reflect the burden of mortality is poorly understood.

**Objective:**

To determine the burden of mortality reflecting community-nominated health risk factors in rural South Africa, where a complex health transition is underway.

**Methods:**

Three discussion groups (total 48 participants) representing a cross-section of the community nominated health priorities through a Participatory Action Research process. A secondary analysis of Verbal Autopsy (VA) data was performed for deaths in the same community from 1993 to 2015 (n = 14,430). Using population attributable fractions (PAFs) extracted from Global Burden of Disease data for South Africa, deaths were categorised as ‘attributable at least in part’ to community-nominated risk factors if the PAF of the risk factor to the cause of death was >0. We also calculated ‘reducible mortality fractions’ (RMFs), defined as the proportions of each and all community-nominated risk factor(s) relative to all possible risk factors for deaths in the population  .

**Results:**

Three risk factors were nominated as the most important health concerns locally: *alcohol abuse*, *drug abuse*, and *lack of safe water*. Of all causes of deaths 1993–2015, over 77% (n = 11,143) were attributable at least in part to at least one community-nominated risk factor. Causes of attributable deaths, at least in part, to *alcohol abuse* were most common (52.6%, n = 7,591), followed by *drug abuse* (29.3%, n = 4,223), and *lack of safe water* (11.4%, n = 1,652). In terms of the RMF, alcohol use contributed the largest percentage of all possible risk factors leading to death (13.6%), then *lack of safe water* (7.0%), and *drug abuse* (1.3%)     .

**Conclusion:**

A substantial proportion of deaths are linked to community-nominated risk factors. Community knowledge is a critical input to understand local health risks.

## Background

Priority setting in health requires robust and timely knowledge on the burden of diseases and mortality to enable efficient resource allocation. While many low- and middle-income countries (LMICs) have built well-functioning civil registration and vital statistics (CRVS) system with good coverage, in many countries, comprehensive systems do not exist [1,2]. There is a drive towards better civil registration system availability and quality globally to ensure better informed decision-making. However, there are fewer voices advocating for the involvement of the community in priority setting. Community members have limited roles in setting local health priorities or even being recognised as active agents who should be consulted in setting the agenda [2–4].

The inclusion of community knowledge is a critical input for health programmes and strategies to support people to address the health challenges they face. Despite the normative support [5], there is ambiguity as to whether community participation in health implies a shift towards local partnership with communities in which power and responsibility are openly negotiated or processes, which manipulate communities into accepting additional responsibilities [6]. Biomedical notions of health are aligned with passive, and even exploitative, forms of participation, while interpretations of health as a human condition are aligned to empowerment-oriented approaches to community participation. Moreover, in the context of COVID-19, many responses have adopted a ‘command and control’ approach, as opposed to bottom-up, community-driven approaches rooted in solidarity and rights for comprehensive public health [7,8].

As described above, incomplete official data can impact decision-making for public services. Strategies to fill data gaps are urgently required. Verbal autopsy (VA) is currently the only realistic alternative to medical certification of deaths in settings where the CRVS system is incomplete or absent. VA is a pragmatic tool to determine the probable cause(s) of death [2,9–11]. Its use has proved sensitive and efficient, providing a valuable method filling the gap in mortality data [10,12].

VA is based on a standardised interview by field workers who collect data on signs, symptoms, and situations preceding death from the final caregiver(s) of the deceased [11]. The World Health Organization (WHO) has a long active role developing and harmonising the practices for VA interviews; WHO 2016 is the latest VA instrument [10,13]. In recent years, VA data have been analysed automatically by computer algorithms, which have proved to produce valid cause of death classifications [14].

In this study, we conducted a secondary analysis of Verbal Autopsy (VA) data to determine the burden of mortality linked to community-nominated risk factors in rural South Africa, where a complex health transition is underway. We sought to provide insights into the connections (or lack thereof) between biomedical concepts of health priorities compared with those put forward by the community.

## Methods

### Study setting

The study was located in South Africa, as part of the VAPAR (Verbal Autopsy with Participatory Action Research, www.vapar.org) programme in which community stakeholders participate in identifying and collectively addressing health challenges in cooperative learning partnerships with the authorities [15]. South Africa is an upper-middle-income country with 66 years of life expectancy in 2019 [16]. There is substantial, entrenched inequality in South Africa in terms of socioeconomic and health status, and access to health services, resulting in a deeply uneven distribution of ill-health and diseases [17].

The study was progressed within the Agincourt Health and Socio-Demographic Surveillance System (HDSS), located in Mpumalanga province, close to the Mozambican border in northeast South Africa [18]. The province is relatively poor and rural, with high unemployment, limited water, sanitation, and electricity services [18,19]. Reflecting on the national situation, the disease burden in the study area is a combination of non-communicable diseases (NCDs), infectious diseases, maternal and child-related disorders with considerably high rate of road accidents and external causes of deaths [20–23], often described as a ‘quadruple burden’ of disease [24]. Age-adjusted HIV prevalence is considerably high in the study area: 19% among men and 26% among women [25].

### Data

#### Identifying community-nominated risk factors

Community-nominated health risk factors were determined by three community discussion groups (total 48 participants) representing rural villages across the Agincourt HDSS. We progressed a Participatory Action Research (PAR) process to identify and address local health concerns. PAR transforms the roles of passive research subjects into active co-researchers and changes agents through collective analysis, taking, and evaluating action and learning from action [26]. We re-engaged participants involved in earlier research across three villages in the Agincourt HDSS [27]. Villages were selected to vary by distance to health facilities and levels of child-headed households, and participants represented a cross-section of the community (traditional healers, community and religious leaders, community health volunteers and family members).

In each village, we held an introductory workshop in which participants nominated a range of issues, collectively validating and prioritising them using ranking and voting. Participants also directed expansion of the participant base to include perspectives that may otherwise be excluded. Each village nominated the highest priority risk factor, hereafter considered as community-nominated risk factor(s). After the nomination, new participants were recruited and worked together, through a series of workshops, sharing, and systematising experiences to build consensus on the problem’s identified, and locally acceptable actions to address them. A total of 16 workshops were held in the common local language *xiTsonga*. Throughout, participants were supported to assume ownership and control of the process. These elements are described elsewhere [28–31].

#### Relating community-nominated risk factors to disease burdens

Longitudinal VA data from 1993 to 2015, for which period the data was available for this analysis, were used to ascertain the probable cause of death of individuals living in the HDSS based upon results from the InterVA-5 algorithm. InterVA-5 assigns each death to up to three cause(s) and the likelihood of that cause [14]. In this analysis, we used the first and most probable cause of death and excluded all causes of death, where the most probable likelihood was <50%.

Using population attributable fractions (PAF) for South Africa extracted from Global Burden of Disease (GBD) data version 2019 [32], we estimated whether each VA classified cause of death was ‘attributable at least in part’ to the community-nominated risk factors. We also calculated the ‘reducible mortality fraction’ (RMF), defined as the *relative* proportions of the community-nominated risk factors contributing to all possible risk factors contributing to deaths. The GBD extraction was performed between 18.1.2021 and 21.1.2021.

PAF is an estimate of the reduction in population mortality if an exposure to a certain risk factor is minimised, as previously described by the WHO [33]. GBD provides PAFs for multiple risk factors for each disease and enables the contribution of individual risk factors to be estimated for any disease. The GBD risk factor hierarchy is based on four levels [34]. Level 1 risk factors are *environmental and occupational, behavioural*, or *metabolic*; Level 2 consists of 20 risks or clusters of risks, such as *air pollution, tobacco, alcohol use, dietary risks, hearing, vision,* and *intellectual disabilities*; Level 3 contains 52 risks or clusters of risks; and Level 4 includes 69 specific risk factors. The causality between these risk-outcome estimates have been previously established [34].

Sex (binary), age at death (continuous), cause of death (CoD) and year of death (continuous) were obtained from the VA data. The main outcomes were (1) the burden of mortality that was attributable, at least in part, in Agincourt to each community-nominated risk factor and (2) the total RMF due to each community-nominated risk factor.

### Procedures and statistical analysis

**Figure 1.**
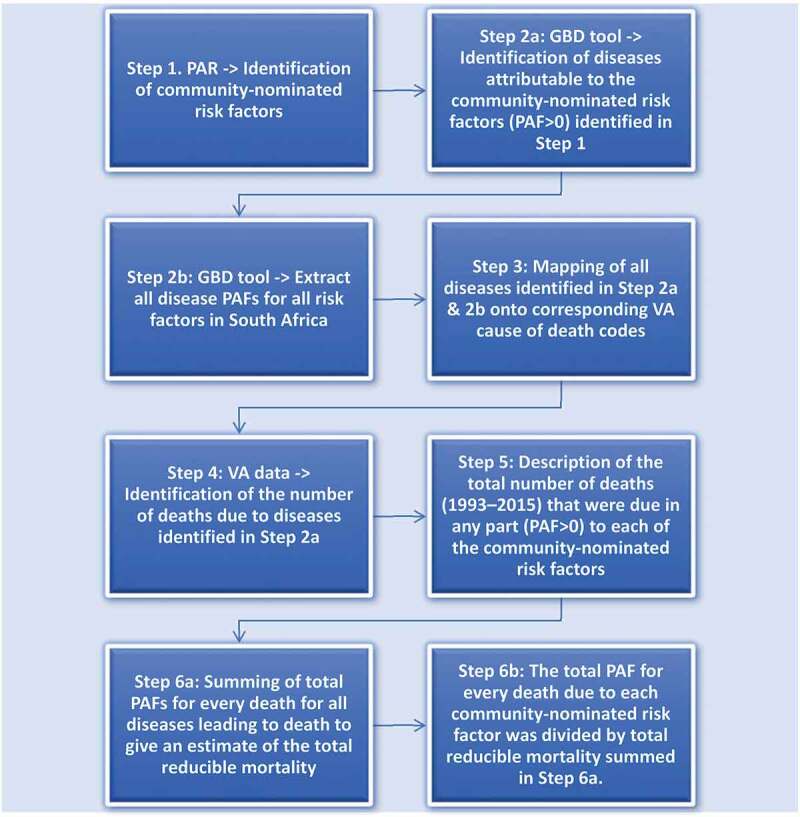
Flowchart of the study phases. (PAR = participatory action research, GBD = global burden of disease, PAF = population attributable fraction, VA = verbal autopsy).

Diseases attributable at least in part to community-nominated risk factors were identified from the GBD data tool, and PAFs from South Africa for all diseases between 1993 and 2015 were extracted covering the same period that VA data were available for this analysis. PAFs were extracted for all ages and both sexes. From these PAFs across all years, the median was used. Diseases for which PAFs were extracted were mapped onto corresponding VA cause of death codes (supplementary material 1). The mapping was based on the WHO Verbal Autopsy Manual’s classification of VA causes of deaths and their corresponding ICD-10 codes. These steps are illustrated in Figure 1.

The total number (and %) of deaths occurring between 1993 and 2015 that were attributable at least in part to each community-nominated risk factor and the number of risk factors that each death was attributable at least in part to were described. Death was categorised as attributable at least in part to a community-nominated risk factor(s) if PAF for the cause of death was >0. If a death was assigned as *Indeterminate*, the PAF was assumed to be 0.

**Figure 2.**
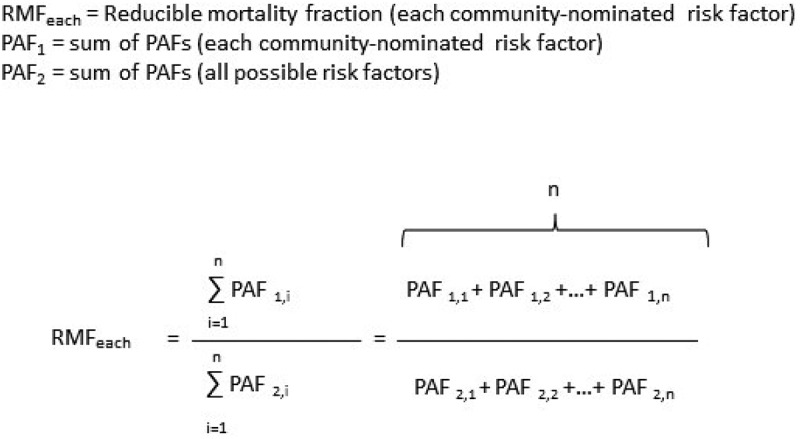
The formula for ‘reducible mortality fraction’: the relative proportion of the PAFs of all risk factors that were due to each and all community-nominated risk factor(s) (example calculation is contained in supplementary material 3). (PAF1 = the PAFs for a community-nominated risk factor for each cause of death multiplied by the number deaths due to that cause, PAF2 = the PAFs for all risk factors for each cause of death multiplied by the number deaths due to that cause, n = 14,430 = the total number of deaths in the population between 1993 and 2015).

To quantify the RMF: the relative proportion of each and all community-nominated risk factor(s) to all possible risk factors contributing to all deaths between 1993 and 2015, we first summed the PAFs of all possible risk factors to every death in the population (Figure 2). Second, we summed the PAFs of each and all community-nominated risk factor(s) to every death. Third, we divided the summed PAFs of each and all community-nominated risk factor(s) with the summed PAFs of all possible risk factors (example calculation is contained in Supplementary material 3). It is possible for PAFs to add up to >100% for any death [35]. However, our aim was not to produce absolute numbers, but rather to ascertain the relative contributions of community-nominated risk factors to deaths in the population.

Results are presented for all community-nominated risk factors and each separately for the whole population and then disaggregated by sex, age category, and mortality category. All deaths and deaths from each risk factor were described according to seven age groups (neonatal (<28 days), infant (28 days–12 months), 1–5 years, 5–15 years, adult (15–50 years), mid-ager (50–65 years) and >65 years), five cause categories as categorised by VA (infectious and parasitic diseases; non-communicable diseases; pregnancy, childbirth, and puerperium-related disorders; neonatal and external causes of death, indeterminate), and over time.

Categorical data were described as n (%); continuous data were described as mean (SD) where normally distributed or median (IQR) where not. The analysis was done using SPSS version 25.

### Ethical considerations

The research was a secondary analysis of VA data from Agincourt HDSS, which has been previously approved by the Committee for Research on Human Subjects at the University of Witwatersrand (Nos. M960720 & M110138). Consent (informed consent at individual and household level as well as community consent from traditional leaders) was secured at the start of surveillance in 1992 and is reaffirmed regularly. The principle of informed consent and right to refusal or withdrawal was fully respected.

## Results

Verbal autopsy data from a total of 14,430 deaths between 1993 and 2015 were assessed using VA (Table 1) after excluding cases where the most probable cause of death was <50% (n = 875). The median (IQR) age at death was 42.4 (26.8, 64.8) years, 6,815 (52.8%) of the deceased were male, and the largest age group adults (15–50 year olds) (n = 6,124, 42.4%). The community-nominated risk factors were (1) alcohol and other drug abuse and (2) lack of safe water. The risk factor *alcohol and other drug abuse* was split into two separate risk factors giving three altogether, all of which were level 2 risk factors, according to the GBD risk factor hierarchy. The GBD data tool provided corresponding risk factors were (1) *alcohol use*, (2) *drug use,* and (3) *unsafe water, sanitation and handwashing*, thus these terms were used hereafter.

**Table 1.** Summary of all deaths and PAFs in terms of alcohol use, drug use, and unsafe water, sanitation, and handwashing according to sex, age, mortality category, and year of death. (RMF = reducible mortality fraction, IQR = interquartile range, N/A = not applicable)

		All deaths (n[%])	Number of causes of deaths attributable at least in part to alcohol use n(%)	Number of causes of deaths attributable at least in part to drug use n(%)	Number of causes of deaths attributable, at least in part unsafe water sanitation and handwashing n(%)	Number of causes of deaths attributable at least in part to alcohol use, drug use, and unsafe water, sanitation or handwashing n(%)	RMF (%) attributable to alcohol use	RMF (%) mortality attributable to drug use	RMF (%) attributable to unsafe water, sanitation and handwashing
Total		14,430 (100)	7,591 (52.6)	4,223 (29.3)	1,652 (11.4)	11,143 (77.2)	13.6	1.3	7.0
Sex	Female	6,815 (47.2)	3,088 (40.7)	2,317 (54.9)	779 (47.2)	5,156 (46.3)	5.1	0.7	3.4
	Male	7,615 (52.8)	4,503 (59.3)	1,906 (45.1)	873 (52.8)	5,987 (53.7)	8.5	0.6	3.5
Age at death	Median (IQR)	42.4 (26.8, 64.8)	50.0 (32.3, 70.8)	41.0 (29.9, 57.6)	17.9 (0.9, 51.6)	43.4 (29.1, 65.0)	N/A	N/A	N/A
Death age groups	Neonate (<28 days old)	371 (2.6)	1 (0.0)	0 (0.0)	0 (0.0)	1 (0.0)	0.0	0.0	0.0
	Infant (28 days–12 months)	866 (6.0)	312 (4.1)	110 (2.6)	480 (29.1)	610 (5.5)	0.3	0.0	2.6
	1–5 years old	869 (6.0)	209 (2.8)	269 (6.4)	261 (15.8)	593 (5.3)	0.3	0.1	1.6
	5–15 years old	402 (2.8)	198 (2.6)	78 (1.8)	75 (4.5)	275 (2.5)	0.4	0.0	0.2
	Adult (15–50 years old)	6,124 (42.4)	3,078 (40.5)	2,332 (55.2)	408 (24.7)	5,088 (45.7)	6.7	0.7	1.1
	Mid-age (50–65 years old)	2,226 (15.4)	1,328 (17.5)	696 (16.5)	152 (9.2)	1,789 (16.1)	2.4	0.2	0.5
	Over 65 years old	3,572 (24.8)	2,465 (32.5)	738 (17.5)	276 (16.7)	2,787 (25.0)	3.6	0.2	0.9
Mortalitycategory*	Infectious and parasitic diseases	6,866 (54.0)	2,993 (39.4)	3,114 (73.7)	1,652 (100)	6,545 (58.7)	7.9	0.9	7.0
	Non-communicable diseases	4,370 (34.4)	3,553 (46.8)	1,052 (24.9)	0 (0.0)	3,553 (31.9)	4.2	0.4	0.0
	Pregnancy-, childbirth and puerperium-related disorders	115 (0.9)	0 (0.0)	0 (0.0)	0 (0.0)	0 (0.0	0.0	0.0	0.0
	Neonatal causes of death	318 (2.5)	0 (0.0)	0 (0.0)	0 (0.0)	0 (0.0)	0.0	0.0	0.0
	External causes of death	1,045 (8.2)	1,045 (13.8)	57 (1.3)	0 (0.0)	1,045 (9.4)	1.5	0.0	0.0
Year of death	1993–1998	1,924 (13.3)	1,059 (14.0)	425 (10.1)	214 (13.9)	1,377 (12.4)	1.9	0.1	1.3
	1999–2004	3,575 (24.8)	1,807 (23.8)	1,121 (26.5)	342 (20.7)	2,783 (25.0)	3.6	0.3	1.7
	2005–2010	5,138 (35.6)	2,588 (34.1)	1,680 (39.8)	550 (33.3)	4,058 (36.4)	4.9	0.5	2.2
	2011–2015	3,793 (26.3)	2,137 (28.2)	997 (23.6)	546 (33.1)	2,925 (26.2)	3.3	0.4	1.8

* Indeterminate cause of death (n = 1,716) was not classified into mortality categories.

There were 63 causes of deaths classifications assigned by VA to the dataset. Of these, 26 (41%) were attributable at least in part to alcohol use, six (11%) to drug use, and two (3%) to unsafe water, sanitation, and handwashing (supplementary material 2). When classified by mortality category, VA causes of deaths that were attributable at least in part to any community nominated risk factor were from the VA mortality categories of infectious and parasitic diseases, NCDs, and external causes of death. None of the deaths were categorised as pregnancy, childbirth, puerperium-related disorders or neonatal causes of death.

**Figure 3.**
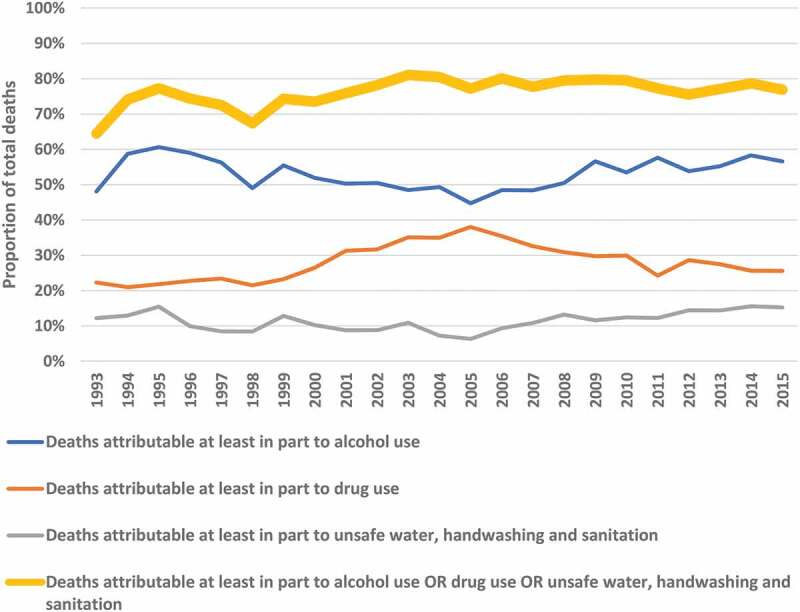
Proportions of total deaths attributable at least in part to alcohol use, drug use, and unsafe water, sanitation, and handwashing or at least one of those, over time (1993–2015). A death was classified as attributable to each one if the population attributable fraction was >0.

Overall, 11,143 (77.2%) deaths were attributable at least in part to at least one community-nominated risk factor; 2,323 (16.1%) to two; and none to all three. The greatest numbers of deaths were attributable at least in part to alcohol use (7,591 [52.6%]), followed by drug use (4,223 [29.3%]) and unsafe water, sanitation, and handwashing (1,652 [11.4%]) (Table 1). The number of causes of deaths attributable at least in part to at least one community-nominated risk factor as a percentage of the total number of deaths were lowest in 1993 and has increased to between 75.0% and 80.0% from 2002 onwards (Figure 3).

For causes of deaths attributable at least in part to alcohol use, the median (IQR) age at death was 50.0 (32.3, 70.8), these deaths occurred in three VA mortality categories; NCDs (46.8%); infectious and parasitic diseases (39.4%); and external causes (13.8%). The proportion of causes of deaths attributable at least in part to alcohol use has been between 50% and 60% from 1993 to 2015.

More females (54.9%) had mortality attributable at least in part to drug use than males; most of these deaths were in the infectious and parasitic diseases (73.7%) or the NCD category (24.9%). The median (IQR) age at death was 41.0 (29.9, 57.6) years (Table 1). Between 1993 and 2005, the proportion of causes of deaths attributable at least in part to drug use almost doubled from 21.0% to 38.0% but has since declined to 25.6%.

Median (IQR) age of causes of death attributable at least in part to unsafe water, sanitation, and handwashing was 17.9 (0.9, 51.6); all of these were in the VA category of infectious and parasitic diseases. Percentages varied between 6.3% and 15.5% from 1993 to 2015, lacking any clear trend.

**Figure 4.**
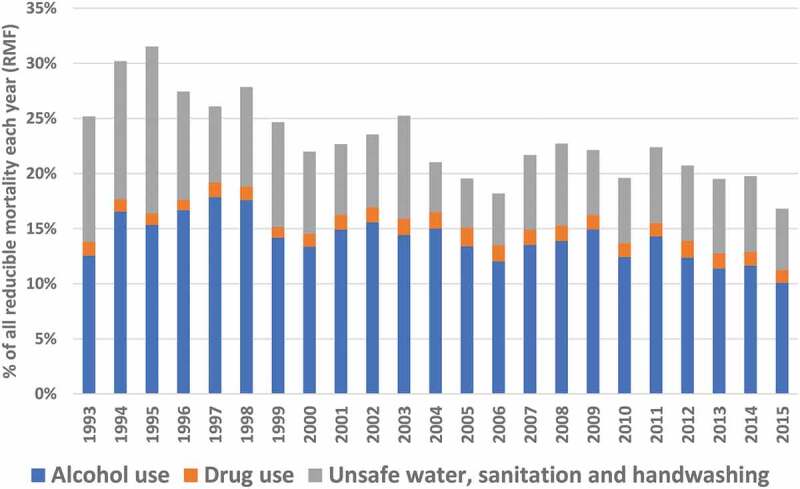
Reducible mortality fractions (RMFs) due to alcohol use, drug use, and unsafe water, sanitation, and handwashing in Agincourt over time (1993–2015).

Of the total PAF of all possible risk factors to all deaths in Agincourt, 21.9% was due to the PAFs of the three community-nominated risk factors; this contribution was greater in males than females (12.6% vs 9.3%) and was largest in the adult age group (8.6%). The contribution of PAFs from alcohol use (13.6%), drug use (1.3%), and unsafe water, sanitation, and handwashing (7.0%) to the PAFs from all possible risk factors, and how that varies over time is shown in (Figure 4).

The proportion of the total PAFs of all possible risk factors that were due to alcohol use was greater in males than females (8.5% and 5.1%, respectively), and greatest in adults (6.7%), >65 year olds (3.6%), and mid-agers (2.4%). These proportions remained stable over time. The proportion of the total PAFs of all possible risk factors that were due to drug use was slightly greater in females (0.7%) than males (0.6%), and greatest in adults, >65 year olds, and mid-agers (0.7%, 0.2%, and 0.2%, respectively). Proportions did not vary between 1993 and 2015. The proportion of the total PAFs of all possible risk factors that were due to unsafe water, sanitation, and handwashing were equal between sexes (Table 1), greatest in infants and children 1–5 year olds, and highest between 2005 and 2010.

## Discussion

The analysis reveals that a substantial number of deaths in this setting can be attributed to community-nominated health risk factors. To a significant degree, the community’s perceptions of the most important local health issues seem to reflect the burden of mortality.

Using both methods, alcohol use was responsible for the largest contribution to mortality. This is consistent with other studies as South Africa has the highest average alcohol consumption in the Southern Africa region [36]. Alcohol has previously been estimated to be the most important risk factor for mortality in the African Region: the alcohol-attributable burden was responsible for 4.7% of all DALYs lost in 2012 [22]. Following global trends, consumption is highly unequal between the sexes: 16.2 L of pure alcohol a year among men and 2.7 L among women [36]. Our analysis demonstrated similar characteristics.

Alcohol plays an important role in external causes of deaths, especially in traffic accidents in South Africa. Between 2010 and 2011, more than 13,000 road traffic fatalities occurred from which 60% involved alcohol use [37]. Thus, when considering the absolute numbers of each mortality category, it seems plausible that a substantial proportion of RMF due to alcohol use was due to external causes of deaths. As with other drugs, alcohol-related harms are highly concentrated among the least affluent in South Africa [38]. Controversially, this group is not usually heard in the process of setting the health priorities and deciding the direction of alcohol legislation [28]. This highlights the legitimacy of community participation, which in turn complements the ideals of collective responsibility and democratic decision-making in core social institutions, such as the health-care system [39].

South Africa is no exception when it comes to tensions in alcohol consumption as an economic versus public health issue [36]. The alcohol industry has considerable lobbying power and uses various tactics influencing different levels of alcohol-related decision-making as well as strongly opposing regulation attempts of reducing overall consumption [40,41]. Given that alcohol markets in Africa have a huge growth potential due to young and increasing population, alcohol use, and abuse are highly relevant public health topics also for the future [42,43].

The COVID-19 pandemic has shown that the situation regarding alcohol regulation is not stationary in South Africa. However, the government introduced new restrictions and temporary sales ban within a considerably short time period, which was a remarkable paradigm shift [44–46], and in contrast to previous slow and stagnated efforts of restriction and regulation policies [40,44,47]. This has opened completely new opportunities for reform, and, importantly, it enables examining the overall health effects of these actions.

In terms of drug use, almost one in six people frequently use illicit drugs in South Africa [48]. In our analysis, the magnitude of the burden of drug use on mortality differed depending on the method used. In particular, deaths categorised as attributable at least in part to drug use were numerous, but when looked at as the proportion of the total PAF contributions to all deaths, drug use had the lowest impact on the RMF. The explanation may be that whilst South Africa has been suffering from a major burden of HIV/AIDS-related deaths [18], drug use was the only community-nominated risk factor causally linked to HIV/AIDS according to GBD. These two facts combined with the low PAF of HIV/AIDS attributable to drug use explain the discrepancy between the relatively high mortality attributable at least in part but low RMF.

Recently, there has been a significant increase in treatment admissions for opioid use disorders – especially due to *nyaope*, a novel low-grade heroin derivative used in rural and poor communities (49,50). An analogous increase in drug use-linked mortality would be hard to detect given that for deaths linked to drug use, the PAFs were very small.

Finally, the least number of deaths was attributed at least in part to unsafe water, sanitation, and handwashing, but its overall contribution to all deaths was intermediate. Nevertheless, our study showed that it was a critical factor in the RMF of young children. South Africa has a high disease burden attributable to the lack of safe and accessible water, and around 5 million citizens do not have a reliable water source [23]. Infrastructure is poorly maintained with an interrupted water supply, which makes the poorest people vulnerable and exposed to discrimination [27,49]. Unsafe water, sanitation, and handwashing are a significant driver of social unrest and disruption, causing many practical problems for daily life and also morbidity, which is not captured in mortality data [50]. Again, it could be seen as a more distal determinant of health, which might be better captured with a community’s view [29,51].

Previously, in South Africa, the estimated burden of mortality due to unsafe water, sanitation, and handwashing was 3.5% of the deaths overall [52], whereas alcohol abuse 6.4% of the overall deaths in sub-Saharan Africa [22]. Although these studies are not directly comparable due to the methodological differences, these estimates support the order and relations of burdens found in this study. According to GBD estimates for South Africa in 2019, the PAF for alcohol use was 5.2%, for unsafe water, sanitation, and handwashing 3.0%, and for drug use 1.1% [53]. Thus, the RMF estimates due to alcohol use and unsafe water, sanitation, and handwashing were higher in our analysis, which might be the reason why they were nominated as high priority risk factors by the community.

This study was concerned with quantifying the burden of mortality according to community-nominated risk factors. We acknowledge, however that the impact of community health priorities extends beyond mortality, which demonstrates only the visible tip of the iceberg [51]. As an illustration from maternal health, approximately 0.3 million maternal deaths are estimated to occur a year [54]. However, about 20 million maternal morbidities, unaccounted for in mortality data, are likely to occur, causing a significant burden of human suffering that the routine mortality data are not able to capture [55]. Unfortunately, the connection between both alcohol and drug use and major disease burdens (e.g. HIV/AIDS and tuberculosis) are not fully recognised by policymakers [41,56], but, in contrast, may be better understood among the community. As part of the wider VAPAR programme, mortality data are supplemented with the community’s perceptions, which, we argue, reflects more explicitly the indirect social consequences – not the mere endpoint of life [31,57].

### Strengths and limitations

This is the first attempt to quantify the mortality burden due to community-nominated risk factors using GBD PAFs that we are aware of. The overall findings seem plausible and consistent when compared with other studies, which is the foremost strength of the study. A further strength was that quantitative and qualitative methods have a complementary capability to provide synergistic benefits to recognise and understand health priorities. Given that VA data is being constantly collected in many settings, this methodology might have wider applicability.

**Figure 5.**
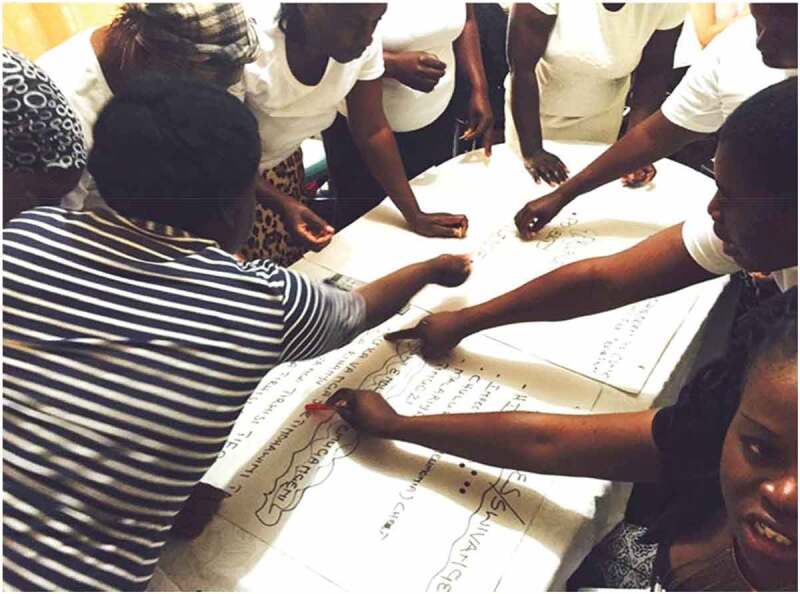
‘Validation through consensus’: nominating and prioritising risk factors was an iterative collective progressed with sensitive facilitation and sustained engagement/dialogue building [Permissions secured for reproduction of image].

In terms of the PAR process through which community-nominated risk factors are identified, our approach built collective understandings of public health priorities faced in rural villages and is designed to enable and strengthen validity and generalisability. At the outset, health concerns nominated by community stakeholders were many. Prioritising was carefully facilitated using consensus-building processes: multiple rounds of ranking, rating, voting, group discussion, appraisal, and collective nomination, ‘validation through consensus’ (Figure 5). This was done iteratively and sensitively over consecutive workshops. Together with collective analysis (of causes and impacts, actions, and taking/evaluating action and learning from action, documented elsewhere [28–31]), ensured the validity and relevance of the risk factors, locally. Participant recruitment supported generalisability.

Participants represented a cross-section of rural villagers with purposive, participant-directed inclusion of additional typically excluded perspectives. As one of the largest and oldest HDSSs in the region, the Agincourt HDSS is an established public health observatory representative of the province and more widely. HDSSs exhaustively cover district-level populations in deprived rural, peri-urban, or urban areas and data extensively conform with other estimates, supporting their generalisability [58]. While nominating one risk factor per village may have been reductive, considering the multitude of entrenched social and health inequalities, our approach progresses consistent presence building alliances and dialogue through a participatory approach identifying local public health priorities that communities deemed to be of the highest importance.

The results should be interpreted with several limitations in mind. There was no complete alignment between GBD terms for risk factors, community-nominated risk factors, and VA and GBD causes of death. Nevertheless, we believe that the terms were close enough for the results to be reliable. Moreover, although VA is proven to be a valid and rigorous method, there is always a chance that some of the causes of deaths are not correctly assigned. This may have led to some imprecision in the results.

Furthermore, some data and linkages between risks and diseases were missing in the GBD data tool [32]. For instance, there were no alcohol use-related PAFs for under five year olds, although children could also be victims in road traffic accidents or interpersonal violence due to alcohol use.

Given that many causes of deaths linked to community-nominated risk factors (HIV/AIDS, pulmonary tuberculosis, acute respiratory infections) were overrepresented in the population, our findings would be slightly smaller if all the ‘Indeterminate’ deaths were distributed evenly to all other VA causes of deaths. Additionally, when unspecified by age and sex, GBD estimates of PAFs for diseases are not entirely precise or available, and for this reason, some of the attributable causes of deaths may be incorrectly classified. We also used PAFs from South Africa, rather than local ones from Agincourt, as PAFs were not available for Agincourt.

Lastly, there were a total of 15,305 VA classified deaths between 1993 and 2015 in the study setting. The size of the population in Agincourt varied between 100,000 and 115,000 persons during the study years, giving a crude death rate of 6 per 1,000 person a year, which is below the South African average of 11.5 between 1993 and 2015 [59]. The reason for this lower-than-average death rate is unknown. The population is well studied, and it is unlikely that deaths are unrecorded. However the fact that they are well studied may mean that they have better health education and lower disease prevalence and death rates than other similar settings in South Africa.

Despite these limitations, we state that the results are reliable given that our aim was not to provide an absolute or accurate number of deaths where a community-nominated risk factor contributed at least in part or the reducible mortality in the population due to community-nominated risk factors. Our aim in this proof-of-concept paper was to use VA and PAF estimates to indicate, for the first time, whether community perceptions of priority risk factors align with scientific estimates of these.

## Conclusions

This study demonstrated that a high proportion of all-cause mortality in rural South Africa is linked to community-nominated risk factors. To a substantial degree, the community’s perceptions of the most important local health issue seem to reflect the burden of mortality. Community’s knowledge is a critical input to understanding local health risks and how they can be addressed. In particular, better recognition of community intelligence and its reflection in statistical data on the burden of disease may complement biomedical approaches with more holistic views, which should be used more regularly.

## Supplementary Material

Supplemental Material

## Paper context

Data on mortality provide valuable information for priority setting but it is sometimes incomplete and underused in local decision-making. Community view seems to reflect the direct mortality burden along with the indirect health effects. When used in collaboration with routine data of mortality, it offers a more holistic approach in setting public health priorities.
