# Supplementary material for: Burden of mortality linked to community-nominated priorities in rural South Africa
Source: Glob Health Action. 2022 Jan 21;15(1):2013599. doi: 10.1080/16549716.2021.2013599 (PMC8786241; doi:10.1080/16549716.2021.2013599)
Supplement: Supplemental Material [file ZGHA_A_2013599_SM6496.docx]

Supplementary material 1. Mapped Verbal Autopsy (VA) and GBD causes of deaths with their population attributable fractions (PAFs) given as medians, between 1993 – 2015.

| Total VA-classified deaths in Agincourt between 1993-2015 | | | Corresponding cause of death GBD codes and total PAFS for all risk factors in South Africa between 1993-2015 | |
| --- | --- | --- | --- | --- |
| VA Code | **N** | **Percent** | **Corresponding GBD code(s)** | **Median PAF** |
| 01.01 Sepsis (non-obstetric) | 16 | 0.1% | Corresponding GBD code not found | - |
| 01.02 Acute respiratory infection including pneumonia | 1214 | 8.4% | Lower respiratory infections | 0.6700 |
| 01.03 HIV/AIDS related death | 3013 | 20.9% | HIV/AIDS | 0.8539 |
| 01.04 Diarrhoeal diseases | 438 | 3.0% | Diarrheal diseases | 0.9294 |
| 01.05 Malaria | 151 | 1.0% | Malaria | Missing |
| 01.06 Measles | 7 | 0.0% | Measles | 0.4335 |
| 01.07 Meningitis and encephalitis | 116 | 0.8% | Meningitis + Encephalitis | 0.0308 |
| 01.09 Pulmonary tuberculosis | 1779 | 12.3% | Tuberculosis | 0.4744 |
| 01.10 Pertussis | 14 | 0.1% | Whooping cough | Missing |
| 01.11 Haemorrhagic fever (non-dengue) | 17 | 0.1% | Corresponding GBD code not found | - |
| 01.99 Other and unspecified infectious diseases | 101 | 0.7% | Leprosy + Sexually transmitted diseases excluding HIV + Other neglected tropical diseases + Trachoma + Rabies + Acute hepatitis (infectious) + Chagas disease + Leishmaniasis + Trypanosomiasis + Schistosomiasis, Cysticercosis, Echinococcosis, Lymphatic filariasis, Onchocerciasis, Ascariasis, Trichuriasis, Hookworm diseases | 0.0723 |
| 02.01 Oral neoplasms | 25 | 0.2% | Lip and oral cavity cancer | 0.6983 |
| 02.02 Digestive neoplasms | 698 | 4.8% | Esophageal cancer + Colon and rectum cancer + Liver cancer + Stomach cancer + Gallbladder and biliary tract cancer + Pancreatic cancer | 0.5318 |
| 02.03 Respiratory neoplasms | 442 | 3.1% | Larynx cancer + Tracheal and bronchus and lung cancer | 0.8096 |
| 02.04 Breast neoplasms | 34 | 0.2% | Breast cancer | 0.2678 |
| 02.05 & 02.06 Reproductive neoplasms MF | 207 | 1.4% | Cervical cancer + Uterine cancer + Ovarian cancer + Prostate cancer + Testicular cancer | 0.4980 |
| 02.99 Other and unspecified neoplasms | 185 | 1.3% | Nasopharynx cancer + Other pharynx cancer + Malignant skin melanoma + Non-melanoma skin cancer + Brain & nervous system cancer + Thyroid cancer + Kidney cancer + Bladder cancer + Mesothelioma + Hodgin lymphoma + Non-Hodgin lymphoma + Multiple myeloma + Leukemia + Other malignant neoplasms | 0.2718 |
| 03.01 Severe anaemia | 4 | 0.0% | Corresponding GBD code not found | - |
| 03.02 Severe malnutrition | 117 | 0.8% | Protein-energy malnutrition | 1.0 |
| 03.03 Diabetes mellitus | 400 | 2.8% | Diabetes mellitus | 1.0 |
| 04.01 Acute cardiac disease | 234 | 1.6% | Ischemic heart disease | 0.9554 |
| 04.02 Stroke | 588 | 4.1% | Stroke | 0.8877 |
| 04.99 Other and unspecified cardiac disease | 543 | 3.8% | Rheumatic heart diseases + Hypertensive heart diseases + Non-rheumatic valvular heart diseases + Cardiomyopathy & myocarditis + Atrial fibrillation & flutter + Aortic aneurysm + Peripheral artery disease + Endocarditis + Other cardiovascular and circulatory diseases | 0.7673 |
| 05.01 Chronic obstructive pulmonary disease | 129 | 0.9% | Chronic obstructive pulmonary disease | 0.7792 |
| 05.02 Asthma | 133 | 0.9% | Asthma | 0.4450 |
| 06.01 Acute abdomen | 146 | 1.0% | Corresponding GBD code not found |  |
| 06.02 Liver cirrhosis | 172 | 1.2% | Cirrhosis and other chronic liver diseases | 0.7013 |
| 07.01 Renal failure | 81 | 0.6% | Chronic kidney disease | 1.0 |
| 08.01 Epilepsy | 50 | 0.3% | Idiopathic epilepsy | 0.2058 |
| 09.01 Ectopic pregnancy | 2 | 0.0% | Ectopic pregnancy | 0.2287 |
| 09.02 Abortion-related death | 8 | 0.1% | Maternal abortion and miscarriage | 0.2291 |
| 09.03 Pregnancy-induced hypertension | 27 | 0.2% | Maternal hypertensive disorders | 0.2286 |
| 09.04 Obstetric haemorrhage | 66 | 0.5% | Maternal hemorrhage | 0.2307 |
| 09.06 Pregnancy-related sepsis | 7 | 0.0% | Maternal sepsis and other maternal infections | 0.2309 |
| 09.07 Anaemia of pregnancy | 2 | 0.0% | Corresponding GBD code not found | - |
| 09.99 Other and unspecified maternal cause of death | 3 | 0.0% | Other maternal disorders + Indirect maternal deaths + Late maternal deaths + Maternal deaths aggravated by HIV/AIDS | 0.2279 |
| 10.01 Prematurity | 21 | 0.1% | Neonatal preterm birth | 1.0 |
| 10.02 Birth asphyxia | 56 | 0.4% | Neonatal encephalopathy due to birth asphyxia and trauma | 0.8815 |
| 10.03 Neonatal pneumonia | 95 | 0.7% | Neonatal sepsis and other neonatal infections | 0.8315 |
| 10.04 Neonatal sepsis | 20 | 0.1% |  |  |
| 10.06 Congenital malformation | 91 | 0.6% | Congenital birth defects | Missing |
| 10.99 Other and unspecified neonatal cause of death | 35 | 0.2% | Other neonatal disorders + Hemolytic disease and other neonatal jaundice | 0.8357 |
| 12.01 Road traffic accident | 421 | 2.9% | Road injuries | 0.2401 |
| 12.02 Other transport accident | 19 | 0.1% | Other transport injuries | 0.2849 |
| 12.03 Accidental fall | 6 | 0.0% | Falls | 0.3861 |
| 12.04 Accidental drowning and submersion | 33 | 0.2% | Drowning | -0.0879 |
| 12.05 Accidental expos to smoke fire & flame | 36 | 0.2% | Fire, heat, and hot substances | 0.1426 |
| 12.06 Contact with venomous plant/animal | 2 | 0.0% | Venomous animal contact | 0.1408 |
| 12.07 Accidental poisoning & noxious subs | 12 | 0.1% | Poisonings | 0.1208 |
| 12.08 Intentional self-harm | 57 | 0.4% | Self harm | 0.2207 |
| 12.09 Assault | 444 | 3.1% | Interpersonal violence | 0.2078 |
| 12.10 Exposure to force of nature | 1 | 0.0% | Exposure to nature forces | 0.0489 |
| 12.99 Other and unspecified external cause of death | 14 | 0.1% | Environmental heat and cold exposure | 0.0777 |
| 98 Other and unspecified NCDs | 182 | 1.3% | Polycystic ovarian syndrome + Urolithiasis + Genital prolapse + Endometriosis + Other gynecological diseases + Urinary tract infections + Other urinary diseases + Acute glomerulodephritis + Musculoskeletal disorders + Skin and subcutaneuous diseases + Gallbladder and biliary diseases + Vascular intestinal disorders + Paralytic ileus and intestinal obstruction + Inflammatory bowel disease + Inguinal, femoral and abdominal hernia + Appendicitis + Other digestive diseases + Gastritis and duodenitis + Peptic ulcer disease + Pneumoconiosis + Other chronic respiratory diseases + Multiple sclerosis + Alzheimers disease and other dementias + Parkinson's disease + Other neurological disorders + Motor neuron disease + Mental disorders + Pancreatitis + Alcohol use disorders + Drug use disorders + Other nutritional deficiencies + Endocrine, metabolic, blood, and immune disorders + Thalassemias + Sickle-cell disorders + G6PD deficiency + Other hemoglobinopathies and hemolytic anemias + Interstitial lung disease and pulmonary sarcoidosis | 0.3505 |
| 99 Indeterminate | 1715 | 11.9% | Corresponding GBD code not found | - |
| Missing | 1 | 0.0% | - | - |
| Total | **14430** | **100.0** |  |  |

Supplementary material 2. Verbal Autopsy (VA) causes of deaths attributable to alcohol use, drug use, and unsafe water, sanitation and handwashing as well as their median PAFs between 1993 – 2015.

| Cause of death (as categorised by VA) | Median PAF of alcohol use | Median PAF of drug use | Median PAF of unsafe water, sanitation and handwashing |
| --- | --- | --- | --- |
| Infectious and parasitic diseases |  |  |  |
| 1.02 Acute respiratory infection, including pneumonia | 0.064 | - | 0.155 |
| 01.03 HIV/AIDS related death | - | 0.025 | - |
| 01.04 Diarrheal diseases | - | - | 0.873 |
| 01.09 Pulmonary tuberculosis | 0.321 | - | - |
| 01.99 Other and unspecified infectious disease | - | 0.001 | - |
| Non-communicable diseases |  |  |  |
| 02.01 Oral neoplasms | 0.469 | - | - |
| 02.02 Digestive neoplasms | 0.139 | 0.013 | - |
| 02.03 Respiratory neoplasms | 0.023 | - | - |
| 02.04 Breast neoplasms | 0.063 | - | - |
| 02.99 Other and unspecified neoplasms | 0.024 | - | - |
| 03.03 Diabetes mellitus | 0.008 | - | - |
| 04.01 Acute cardiac disease | 0.003 | - | - |
| 04.02 Stroke | 0.085 | - | - |
| 04.99 Other and unspecified cardiac disease | 0.070 | - | - |
| 06.02 Liver cirrhosis | 0.664 | 0.121 | - |
| 08.01 Epilepsy | 0.206 | - | - |
| 98 Other and unspecified noncommunicable diseases | 0.023 | 0.009 | - |
| External causes of death |  |  |  |
| 12.01 Road traffic accident | 0.095 | - | - |
| 12.02 Other transport accident | 0.102 | - | - |
| 12.03 Accidental fall | 0.065 | - | - |
| 12.04 Accidental drowning and submersion | 0.054 | - | - |
| 12.05 Accidental exposure to smoke, fire and flames | 0.060 | - | - |
| 12.06 Contact with venomous animals and plants | 0.058 | - | - |
| 12.07 Accidental poisoning and exposure to noxious substance | 0.049 | - | - |
| 12.08 Intentional self-harm | 0.206 | 0.055 | - |
| 12.09 Assault | 0.142 | - | - |
| 12.10 Exposure to force of nature | 0.049 | - | - |
| 12.99 Other and unspecified external cause of death | 0.077 | - | - |


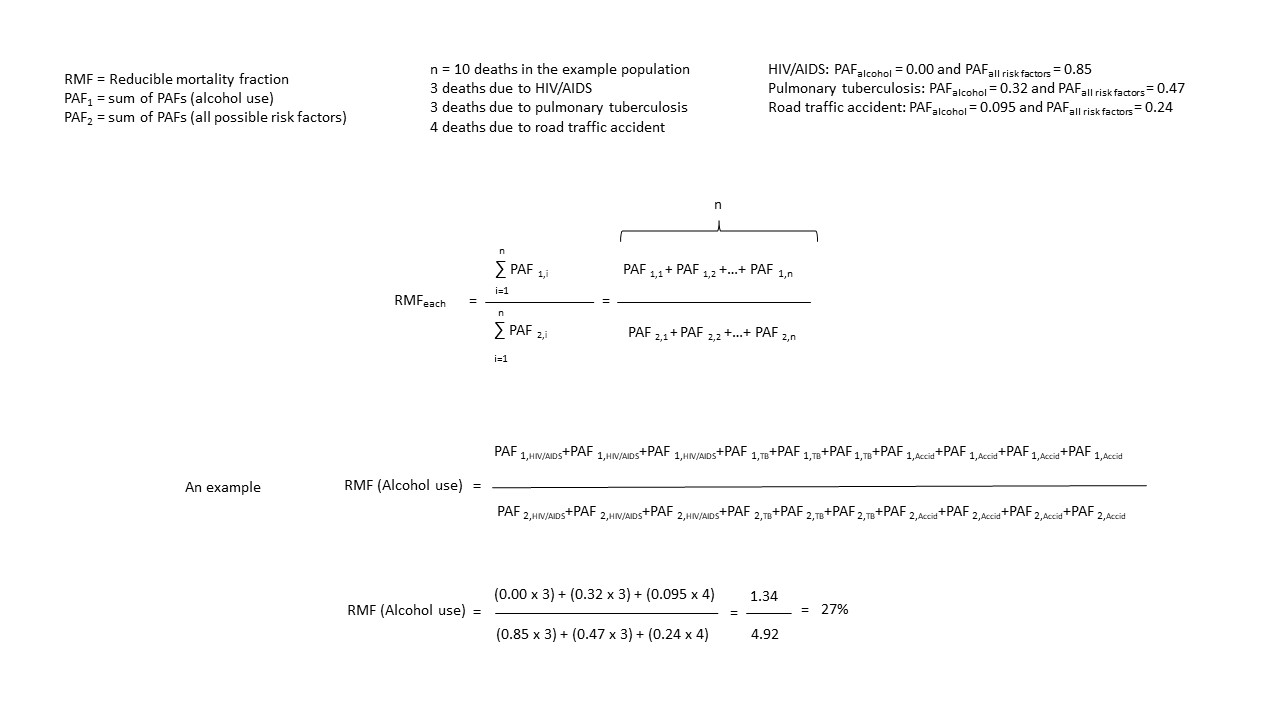


Supplementary material 3. An example calculation of reducible mortality fraction (RMF) for alcohol use.
